# Wetland Park Planning and Management Based on the Valuation of Ecosystem Services: A Case Study of the Tieling Lotus Lake National Wetland Park (LLNWP), China

**DOI:** 10.3390/ijerph20042939

**Published:** 2023-02-08

**Authors:** Lu Yang, Zhi Zhang, Weikang Zhang, Tong Zhang, Huan Meng, Hongwei Yan, Yue Shen, Zeqian Li, Xiaotian Ma

**Affiliations:** 1 Landscape Planning Laboratory, Forestry College, Shenyang Agricultural University, Shenyang 110866, China; 2 Liaoning Panjin Wetland Ecosystem National Observation and Research Station, Shenyang 110866, China

**Keywords:** ecosystem services valuation, land-use, planning and management, Lotus Lake National Wetland Park (LLNWP)

## Abstract

The valuation of wetland ecosystem services and the construction of environmental landscapes are generally recognized as contributing to the sustainable development of human wellbeing. The valuation of ecosystem services plays an important role in planning for the recovery of degraded wetlands and in urban wetland park management; however, the role of the valuation of ecosystem services is always ignored. To bring more intuitive awareness to the importance of the ecological functions of wetlands and to rationally plan wetland parks, the Lotus Lake National Wetland Park (LLNWP), an urban wetland park in Northeast China, was selected as the study area. We referred to the millennium ecosystem assessment (MA) method and calculated the valuation of this park using the market value, benefit transfer, shadow engineering, carbon tax, and travel cost. ArcGIS was used for remote sensing interpretation. The research results were as follows. LLNWP was classified under seven types of land-use. The functions of the ecosystem services included provisioning, regulating, supporting, and cultural services, and their total value in LLNWP was 11.68×10^8^ CNY. Regarding the per-unit area value of the ecological service functions of different land types, it was found that forest swamp > herbaceous swamp > artificial wetland > permanent river > floodplain wetland. Combined with the characteristics of the functions of its ecosystem’s services, LLNWP was divided into ecological and socio-cultural functions. Then, according to the main service functions of the different land types, we propose that the space in LLNWP can be reused, and proposal planning and management suggestions can be made with the aim of preserving the basic functions.

## 1. Introduction

Wetlands offer unique resources that provide irreplaceable functions of ecosystem services to inhabitants in the transition zone between land and water systems. Their ecosystems supply essential substance, energy, and information to human society [1], supporting human wellbeing directly or indirectly [2]. The functions of global ecosystem services can be divided into 17 aspects [3]. Wetlands provide USD 47 trillion worth of ecosystem services yearly [4], which represents more than forests or grasslands [5]. However, the overuse and undervaluation of wetlands cause wetland loss, further increasing fragmentation and seriously reducing their ecological functions [6]. Despite the availability of some data on global ecosystem services, the value of wetlands has still not been recognized by policymakers to a large extent. Due to the lack of evaluation of the functions of wetland ecosystem services, people do not realize the value of wetlands and lack awareness regarding wetland protection. Wetland valuation is an available policy tool for policymakers and environmental planners to justify the costs of wetland preservation activities [7]. Thus, relevant researchers urgently need more targeted data and information. 

Wetlands are declining in area and quality and are thus in serious trouble and under mounting pressure [8]. As people have continuously placed demands on the ecosystem, the global wetland landscape has been destroyed, and the area of wetlands has continued to decrease. According to the Global Wetland Outlook [5], the rate of wetland disappearance has reached three times that of forested areas; between 1970 and 2015, wetlands declined by approximately 35%. Since the 18th century, 87% of wetlands have disappeared. The main driving factors of wetland degradation are drainage, conversion, pollution, and species invasion. This degradation has led to decreased biodiversity, damage from critical environmental pollution, and the endangered species crisis, which gravely affects the ecological environment and directly threatens global sustainable development. The valuation of wetland ecosystem services can raise people’s wetland protection awareness, and the evaluation results can also guide wetland landscape planning and design to restore and protect existing wetlands. Presently, the majority of research on wetlands has been independent of the valuation of ecosystem services and the planning and management of wetland parks. Although some wetland designs are based on landscape ecology, this still needs to be further explored in terms of the value of the functions of ecosystem services. Wetland landscape planning can be associated with an ecosystem’s functional values through the use of land space [9].

Wetland parks [10] are an effective way of solving the contradiction between wetland development and protection via the development of eco-tourism. They have the functions of improving the urban heat island effect, purifying water quality, and regulating the local climate [11,12]. Wetland parks vary in size, and small wetlands are of great significance to the urban ecological environment. The defined area of wetland parks generally ranges from 0.1 hm^2^ to dozens of hm^2^ and generally does not exceed 8 hm^2^ [13]. They provide important ecosystem services such as stepping stones for biological migration, creating beautiful urban landscapes [14], etc.

**Figure 1:**
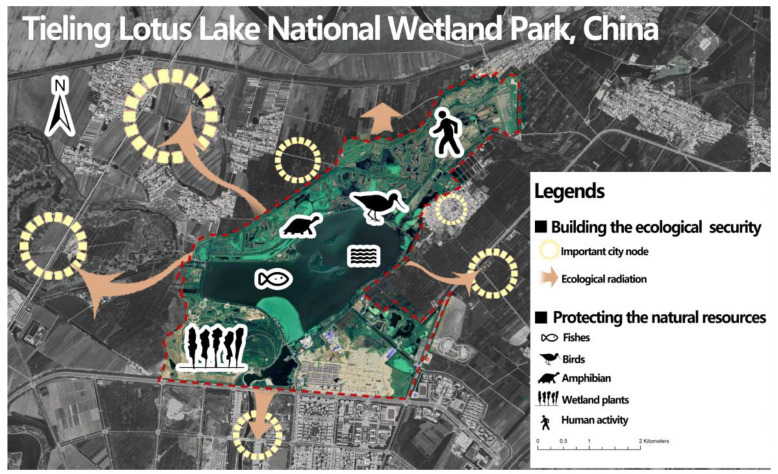
A schematic diagram of the Lotus Lake National Wetland Park’s (LLNWP) ecological role.

Lotus Lake National Wetland Park (LLNWP) is located between the new and old towns, protecting the ecological security of Tieling City, improving the investment environment, and driving the local economy of Tieling City. It is located on the Northeast Asia–Australia bird migration route and is rich in bird resources. LLNWP provides a habitat for many key nationally protected birds and rare and endangered birds, maintaining international species diversity [15]. LLNWP has an irreplaceable role in protecting natural resources, the ecological environment, and ecological security (Figure 1). This study aims to establish the relationship between ecosystem services and landscape planning through land space, strengthen the application progress of ecosystem services in landscape planning research, and highlight the direction of future wetland park planning and management research.

## 2. Materials and Methods

### 2.1. Study Area

**Figure 2:**
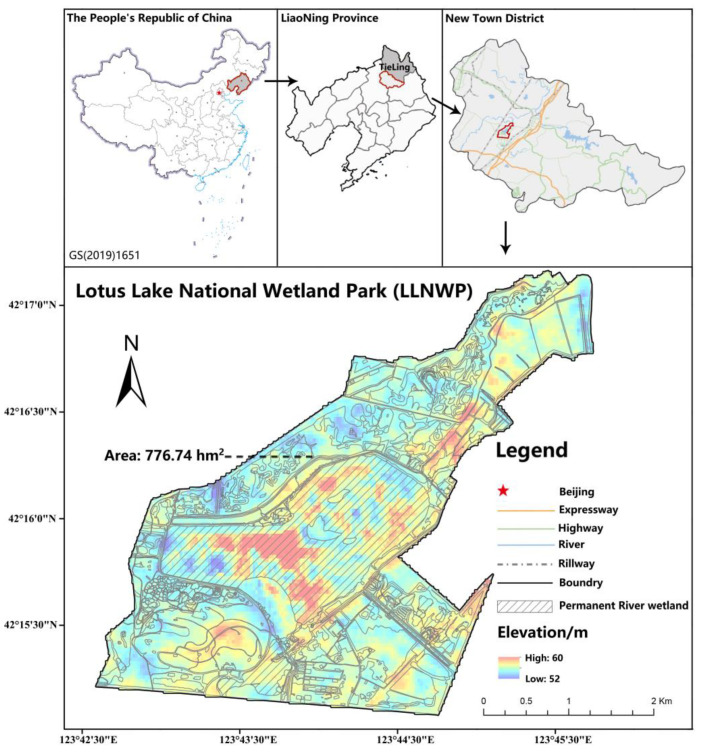
Location of Tieling LLNWP.

LLNWP (123°41′~123°48′ E, 42°15′~42°18′ N) is an urban wetland park that is located in the south of Northeast China, close to Shenyang City. This is a location that is well-known in China for its old industrial base, located at the junction of the Liao River, Fan River, and Chai River. The study area was 776.74 hm^2^ (Figure 2). The climate is a temperate continental monsoon climate, with an average annual temperature of 6.3 °C, average annual precipitation of 700 mm, an annual sunshine duration of 2801 h, and a frost-free period of 127–165 days. The wetland is located on the alluvial plain of the Liao River and the first terrace of the Chai River, with flat terrain and an altitude of 50.5 m~61.8 m. The topography is composed of alluvial plains–floodplains–flat depressions in the low terraces, and the soil consists of meadow soil and paddy soil. Water resources are abundant, and most groundwater levels are between 1.0 m and 3.0 m. LLNWP is mainly comprised of natural wetlands combined with the restoration and reconstruction of artificial wetlands. The northeast and southern areas mainly consist of artificial wetlands, and the remaining areas are comprised of natural wetlands. The natural wetland types are mainly freshwater river wetlands and swamps, and the artificial wetlands are mainly paddy fields and reservoir ponds. The water flow is mainly horizontal, but there will be some vertical water flow changes due to miniature terrain drops. LLNWP has a total of 9.1 million aquatic plants of 26 species, including reeds, cattails, and water onions. The main vegetation in the natural wetland is reeds, lotus, wild rice stems, etc. In the northeast artificial wetland, the main vegetation types are reeds and rice; the southern right side mainly includes landscape plants, including calamus, cattails, etc., and the southern left side is dominated by rice plants. As the main function of the artificial wetland is to restore the original degraded natural wetland, LLNWP contains less porous materials of three main types: limestone, zeolite and crushed stone. This area possesses crucial value in providing animal and plant habitats and breeding grounds, maintaining species diversity, and ensuring urban ecological security.

### 2.2. Data Sources 

The study data were derived from the observation data of the ecosystem fixed station in the wetland park, the field survey sampling, and late laboratory measurement data obtained on 11 September 2021. The remote sensing image interpretation was undertaken in 2021, and other data referred to the China Economic and Social Development Statistics Database, Tieling City Natural Resources Affairs Services Center and *Tieling City’s Statistical Yearbook, 2020*, etc. This included data relating to land-use, substance production, water purification., water conservation, carbon sequestration and oxygen release, climate regulation, biodiversity protection, soil retention, and entertainment and education. All ecosystem services valuation data were entered in Excel 2010, including data type, data category, value, and data source, as shown in Table 1 below. In the following calculations, some conversions to the International System of Units were required. For example: 1 hm^2^ = 10^4^ m^2^, 1 t = 10^3^ kg, 1 kW·h = 3.6 MJ = 3.6 × 10^6^ J.

### 2.3. Ecosystem Services Valuation Methods 

The research referred to the evaluation system proposed by the millennium ecosystem assessment (MA), Costanza, in addition to the domestic main research results of wetlands, combined with the main characteristics of the LLNWP ecosystem and ecological evolution [21]. According to the characteristics of different ecological service functions, the LLNWP ecosystem was divided into four main categories: supporting services, regulating services, provisioning services, and cultural services. According to the different types of Lotus Lake wetland ecosystem services, the market value, benefit transfer, shadow engineering, carbon tax, and travel cost were calculated. Finally, the evaluation index system [22,23] of the LLNWP ecosystem services function value was determined (Table 2).

### 2.4. Calculations and Data Analysis

#### 2.4.1. Provisioning Services

(1) Substance Production

The wetland ecosystem provisioning services mainly include directly usable water, aquatic products, and other intuitive resources. The LLNWP mainly provides drinking water, farmland irrigation, and wetland products such as reeds, lotus roots, rice, and other products for nearby residents. Therefore, according to the market value method [22], the equation is as follows:

(1) $$V_{1}=\sum Q_{m}\times D_{m}\times J_{m}$$

where *V*_1_ represents the annual value of the substance product (CNY/a); *D_m_* represents the annual output per unit area of the m substance (t/hm^2^·a); *Q_m_* represents the production area of the m substance (hm^2^); and *J_m_* represents the market price of the m substance (CNY/t).

According to field surveys, the annual yield of the rice unit area of Tieling City is 8.6249 t/hm^2^·a. The rice field area of the LLNWP is 94.54 hm^2^, the reed area is 23.7 hm^2^, and its annual yield per unit area is 10.3124 t/hm^2^·a. In addition, the lotus root area is 88.21 hm^2^, its annual yield per unit area is 48.749 t/hm^2^·a, and the water area is approximately 208.97 hm^2^. The price of rice, reed, lotus, and water is 2830 CNY/t, 600 CNY/t, 3000 CNY/t, and 3.2 CNY/t, respectively.

#### 2.4.2. Regulating Services

(1) Water Purification

The wetland plants of the LLNWP guarantee the quality of its water resources. The degradation of microorganisms and matrix adsorption can purify water and reduce the damage caused by sediment and pollutants. According to the shadow engineering method, the cost of wetland water purification can be replaced by the cost of sewage treatment in an urban city. The equation is as follows [24]:

(2) $$V_{2}=Q\times L$$

where *V*_2_ represents the annual value of wetland water purification function (CNY/a); *Q* represents the annual amount of sewage purified by the wetland (t/a); and *L* represents the unit sewage treatment cost (CNY/t).

The LLNWP’s water purification function treats urban regenerated water at 50,000 tons per day. Due to the higher latitude, the frost period is longer each year. Water purification services cannot be provided during the frozen period, so this paper calculated the value of water purification during the 165-day frost-free period each year. Furthermore, 825,000 tons of water are purified each year, and the wastewater treatment cost is 2.73 CNY/t.

Water quality detection indicators mainly include chemical oxygen demand, COD (mg/L); total phosphorus, TP (mg/L); ammonia nitrogen content, NH_4_^+^ (mg/L); and dissolved oxygen, DO (mg/L). Based on field measurements undertaken in September 2021, the initial average COD concentration at the water inlet of the LLNWP was 23.88 mg/L, which meets the national IV water standard, and the COD removal rate of the water outlet after purification was 18.2%. The initial average TP concentration was 0.125 mg/L, which accords with the national Class III water standard; furthermore, the TP concentration at the water outlet was lower than the national Class II water standard, so the LLNWP had a high removal rate of total phosphorus, reaching 85.7%. The initial average NH_4_^+^ concentration in the park was 0.46 mg/L, and the removal rate was 87.68%. The NH_4_^+^ concentration of the water body was lower than the national Class II water standard. The initial average DO concentration in the LLNWP was 2.84 mg/L, which belongs to the national standard for Class V water. The DO concentration at each water outlet slightly increased, but there was no significant change. On the whole, the water purification degree of the LLNWP was improved.

(2) Water Conservation

The LLNWP has the functions of water storage and flood regulation. It can provide its own stored water resources to surrounding areas during droughts. When floods occur, it first stores water resources and then absorbs and filters the water resources. Using the shadow engineering method to measure the ecological benefits of water conservation is more convenient than direct measurement, which is also difficult. It has become a common method for calculating flood regulation and water storage valuation in ecosystems. The ecological benefit of wetland flood regulation and water storage is expressed by the product of the unit water storage cost and the total wetland water storage [25,26]. The equation is as follows:

(3) M=n×p

where *M* represents the annual value of water conversation (CNY/a); *p* represents the annual investment cost for every 1 m^3^ of storage capacity invested (CNY/m^3^·a); and *n* represents the amount of water storage (m^3^).

The LLNWP’s current river wetland area is 208.97 hm^2^, and the average water level line is 54 m; therefore, the amount of water storage is 112.84 × 10^6^ m^3^. The water storage cost is 0.67 CNY/m^3^·a.

(3) Carbon Fixation and Oxygen Release
  ① Wetland Marsh Vegetation:

Wetland plants and crops need to photosynthesize every day, so absorbing carbon dioxide and releasing oxygen can generate carbon sequestration value. Estimating the carbon sequestration value first requires calculating the biomass of wetland plants and crops. Biomass is also called natural plant net primary production (NPP), which refers to the amount of organic dry matter accumulation by plant communities, in unit time and area, through photosynthesis under natural environmental conditions [27]. The usual calculation method of NPP is obtained by combining the Chikugo model with plant physiological ecology and statistical methods [28]. The equation is as follows:

$$NPP=0.29\exp\left[-0.216{\left(RDI\right)}^{2}\right]\times \frac{R_{n}}{4.2}\times 0.01$$

$$RDI=\frac{R_{n}}{LR}$$

$$R_{n}=0.35R_{z}$$

(4) L=2507.4−2.39T

where *NPP* represents the net primary productivity of plants (g/m^2^·a); *RDI* is radiant dryness (g/m^3^); *R_n_* represents the net amount of radiation received on the land surface (J/cm^2^·a); *R_z_* represents the total solar radiation in Tieling City (J/cm^2^·a); *L* represents the latent heat of evaporation (J/g); *R* represents the annual precipitation (mm/a); and *T* represents the annual average temperature (°C).

It can be seen from Table 1 that the total solar radiation in Tieling City is 50.49 × 10^4^ J/cm^2^·a, the annual precipitation is 692 cm/a, and the annual average temperature is 8.75 °C.

This calculation first estimates the yield of the surface plant of the LLNWP to calculate the swamp plants’ carbon fixation amount and then uses the plant photosynthetic equation: 6CO_2_ + 6H_2_O → C_6_H_12_O_6_ + 6O_2_ → polysaccharide. According to the conservation of quality relationship, 162 g dry matter of the surface plant should absorb 264 g CO_2_; that is, 1.63 t CO_2_ can be converted into 1 t dry matter. We can infer that 1 t dry matter can fix 0.44 t carbon. Therefore, the dry matter fixation amount on the ground multiplied by 0.44 t is equal to the total plant carbon fixation amount on the ground, and fixed 0.44 t carbon can release 1.2 t O_2_. The dry matter fixation amount on the ground multiplied by 1.2 t is equal to the total oxygen release of the plant. The equation is as follows:

$$Q_{CO_{2}}=0.44N\times S$$

(5) $$Q_{O_{2}}=1.2N\times S$$

where *Q_CO_2__* represents the total annual carbon sequestration (t/a); *Q_O_2__* represents the total annual amount of oxygen released (t/a); *N* represents the annual net production of plants per unit area (t/hm^2^·a); and *S* represents the wetland area covered by vegetation (hm^2^).

Substituting into Equation (4), the net primary productivity (NPP) of marsh plants is 9720 g/m^2^·a, that is, the annual net production of aboveground swamp plants per unit area of wetland is 97.2 t/hm^2^·a. Combined with the swamp plant area of 230.88 hm^2^ in the LLNWP, it can be concluded that the fixed dry matter amount of the swamp plants is 2.24 × 10^4^ t. Therefore, the annual total aboveground carbon sequestration of swamp plants is 9874.28 t/a; the annual total aboveground oxygen release is 26,929.85 t/a.

  ② Wetland Crops:

A field investigation showed that rice was the main crop in the LLNWP. Therefore, this study calculated the carbon fixation and oxygen release of crop biomass through the moisture content of the crops and the plant economic coefficient. The plant economic coefficient *K* is the ratio of the economic yield to the biological yield of the crops, which is directly affected by planting technology, soil conditions, the natural environment, and other factors [29]. The equation is as follows:

(6) $$Q_{i}=D\times \frac{\left(1-P\right)}{K}$$

where *Q_i_* represents the annual aboveground biomass of crops (t/a); *D* represents the annual economic output of crops (t/a); *P* represents the moisture content of crops; and *K* represents the economic coefficient.

According to laboratory calculations, the economic coefficient of rice is between 0.35 and 0.6, so the average value is 0.47. It can be seen from Table 1 that the standard grain water content percentage of rice is 13.5. Since the annual output of rice per unit area in Tieling City is 8624.9 kg/hm^2^·a, and the area of the LLNWP rice fields is 94.54 hm^2^, the annual economic output of LLNWP rice is 815.4 t/a.

The underground rice biomass of the LLNWP can be calculated according to the average underground biomass per unit area [30], and the equation is as follows:

(7) $$Q_{j}=m\times n$$

where *Q_j_* represents the annual biomass of underground crops (t/a); *m* represents the annual average underground biomass per unit area (g/m^2^·a); and *n* represents the planting area of crops (hm^2^).

It can be seen from Table 1 that the average annual underground biomass per unit area of rice is 55 g/m^2^·a. The paddy field area of the LLNWP is 94.54 hm^2^. According to Equation (7), the biomass *Q_j_* of underground rice crops is calculated to be 52 t/a.

According to Equation (4), the annual net crop production per unit area of the LLNWP is 9.64 t/hm^2^·a. The plant photosynthetic equation is: 6CO_2_ + 6H_2_O → C_6_H_12_O_6_ + 6O_2_ → polysaccharides. According to Equation (5), the carbon fixation amount of crops on the ground is 401.15 t/a, and the oxygen release amount is 1094.05 t/a. The carbon content coefficient of rice is 0.47. Therefore, the carbon fixation amount of crops underground is the crop plants’ biomass carbon-containing coefficients multiplied by the underground biomass, which equals 24.44 t/a.

  ③ The Valuation:

According to the photosynthesis equation, plants fix CO_2_ and release O_2_. In this study, the carbon tax method and afforestation cost method [31] were used to evaluate the value function of ecosystem carbon fixation and oxygen release. The equation is as follows:

$$V_{CO_{2}}=Q_{CO_{2}}\times Y_{1}$$

(8) $$V_{O_{2}}=Q_{O_{2}}\times Y_{2}$$

where *V*_*CO*_2__ represents the annual carbon sequestration value of the wetland (CNY/a); *Y_1_* represents the value of CO_2_ (CNY/t); *V*__*O*_2_ represents the annual oxygen release value of the wetland (CNY/a); *Y*_2_ represents the O_2_ value (CNY/t); *Q_CO_2__* is the annual total amount of carbon sequestration in the LLNWP (t/a); and *Q_O_2__* is the annual total amount of oxygen released in the LLNWP (t/a).

The carbon tax rate of Sweden is commonly used internationally. China is currently a developing country, so the carbon tax rate of Sweden commonly used internationally is slightly higher than that of China. Therefore, the common international values cannot be used directly when estimating the value of carbon sequestration. The carbon tax rate was calculated at the average value of the international standard of 150 SEK/t and the afforestation cost in China of 250 CNY/t; 150 SEK/t was converted to an RMB price of 969 CNY/. Consequently, the carbon tax was calculated to be 609.5 CNY/t. The product of the amount and cost of carbon fixation can be used to calculate the total value of CO_2_ fixation. The unit cost of industrial oxygen production is 0.4 CNY/kg.

(4) Climate Regulation
  ① Temperature Regulation:

Wetlands can improve the climatic environment in a certain area, increase the humidity of the air, interact with the surrounding environment through processes such as water vapor evaporation, and bring benefits to air quality. According to the benefit transfer method, air conditioning was selected as a replacement for temperature regulation. The value of wetland temperature regulation is equal to the electricity cost of air conditioning to reduce the same temperature [32]. The equation is as follows:

(9) $$V_{c}=\frac{Q}{3.6e}\times C\times T\times S$$

where *V_C_* is the annual value of wetland temperature regulation (CNY/a); *e* represents the air-conditioning energy efficiency ratio; *Q* represents the heat absorbed per unit area of wetland (MJ/d·hm^2^); *T* represents the frost-free period (d/a); *C* represents the local electricity rate standard (CNY/kW·h); and *S* represents the wetland area (hm^2^).

The unit area of wetland per day can absorb 81.8 MJ from the environment in the summer, which is equivalent to 189 air conditioners cooling all day. The average energy efficiency ratio of air conditioners is 3.4, the freezing period is 200 days each year, the electricity bill is 0.5 CNY/kW·h, and the wetland area is 534.39 hm^2^.

  ② Humidification:

The evaporation of wetland water resources can increase air humidity, so humidifiers are used as the humidification function’s replacement. This is replaced by the more common household humidifiers on the market with a power of 32 W, and the function value is calculated using the electricity cost consumed by the humidifier. According to the shadow engineering method [25], the equation is as follows:

(10) $$V_{h}=Q\times t\times C$$

where *V_h_* represents the annual value of wetland humidification (CNY/a); *t* represents the electricity consumption to convert a unit volume of water into water vapor (kW·h/m^3^); *Q* represents the amount of water evaporated in the wetland (m^3^/a); and *C* represents the local electricity bill standard (CNY/kW·h).

The electricity consumption required by the humidifier to convert 1 m^3^ water into vapor is approximately 125 kW·h/m^3^; the average electricity price is 0.5 CNY/kW·h in Tieling City. The average annual evaporation is 1262 mm in Tieling City, and the total area of the LLNWP is 776.74 hm^2^; therefore, the annual evaporation of wetland water is 9.8 × 10^6^ m^3^/a.

(5) Soil Retention

The function of wetland soil retention is mainly to conserve the soil, thereby reducing soil erosion. According to the shadow engineering method [25], 1 kg of diammonium phosphate fertilizer and organic fertilizer can provide the value of nitrogen, phosphorus, and organic matter quality to replace the calculation of wetland soil retention value. The equation is as follows:

(11) $$V_{8}=Q\left(\frac{PC_{1}}{R_{1}}+\frac{NC_{1}}{R_{2}}+MC_{2}\right)$$

where *V*_8_ represents the annual value of wetland soil retention (CNY/a); *Q* represents the annual amount of soil retention (kg/a); *P* represents the average phosphorus content of the soil (g/kg); *N* represents the average nitrogen content of the soil (g/kg); *M* represents the content of soil organic matter (g/kg); *R*_1_ represents the phosphorus content of diammonium phosphate fertilizer (g/kg); *R*_2_ represents the nitrogen content of diammonium phosphate fertilizer (g/kg); *C_1_* represents the price of diammonium phosphate fertilizer (CNY/kg); and *C*_2_ represents the price of organic matter (CNY/kg).

The nitrogen content of diammonium phosphate fertilizer *R*_2_ is 180 g/kg; the phosphorus content of diammonium phosphate fertilizer *R*_1_ is 460 g/kg; the price of organic matter *C*_2_ is 0.32 CNY/kg; and the price of diammonium phosphate fertilizer *C_1_* is 2.1 CNY/kg. The average soil nitrogen content is 1.16 g/kg, the average soil phosphorus content is 0.05 g/kg, and the soil organic matter content is 2.01 g/kg. Furthermore, soil retention is equal to soil erosion minus sediment production.

#### 2.4.3. Supporting Services

(1) Biodiversity Protection

According to the planning text of the LLNWP and the survey of the Tieling Natural Resources Affairs Service Center, there are 294 species of animals in the LLNWP. Among them, vertebrates include 223 species of birds, 20 species of fish, 6 species of amphibians, and 14 species of mammals. There are 31 species of invertebrates (including benthos and zooplankton).

  ① Bird Conservation:

The bird resources in the LLNWP are relatively rich. There are 223 species of birds in 16 orders and 48 families. These include 21 species of raptors, accounting for 9.4% of the total; 48 species of wading birds, accounting for 21.52%; 39 species of swimming birds, accounting for 17.49%; and 115 species of other forest birds, accounting for 51.57%. According to the long-term monitoring by the monitoring station of the LLNWP, due to the high latitude of Tieling City, the national first-level and second-level protected birds all nest and breed in the LLNWP from March to July, and they migrate south for the winter from August to November. According to the market value method [22], the equation is as follows:

(12) $$V_{AC}=\sum_{i=1}^{n}J_{i}Q_{i}$$

where *V_AC_* represents the annual value of bird conservation (CNY/a); *Q_i_* represents the annual number of wild protected birds at the *i*-th level, *i* = 1 represents first-level national conservation, and *i* = 2 represents second-level national conservation (pcs/a); and *J_i_* represents the market price of the *i*-th level of wild protected birds (CNY/pcs).

During the observation period of a year, 17 different first-level national conservation bird species were observed, including *Ciconia boyciana*, and 69 different second-level national conservation bird species were observed, including *Egretta garzetta*. According to the questionnaire on the “willingness to pay for bird protection” [33] of residents in 2021 in the Tieling area, the price of national first-level protected birds is 13.51 × 10^4^ CNY per bird; the price of national second-level protected birds is 17.41 × 10^4^ CNY per bird.

  ② Habitat:

According to the benefit transfer method, the wetland habitat value can be estimated by the wetland ecosystem biodiversity conservation unit value [34], the equation is as follows:

(13) $$V_{h}=S\times I$$

where *V_h_* represents the annual value of wetland habitat (CNY/a); *S* represents the habitat area; and *I* represents the annual unit value of wetland ecosystem biodiversity protection (CNY/hm^2^·a).

According to the Statistics Information Network of Liaoning Province. (https://tjj.ln.gov.cn/, accessed on 25 July 2021), the unit value of wetland ecosystem biodiversity protection is 2212.2 CNY/hm^2^·a. Moreover, the habitat area is 703.47 hm^2^.

#### 2.4.4. Cultural Services

The cultural service function mainly refers to the entertainment and education functions provided by the wetland ecosystem, mainly including tourism and leisure, culture, education, and scientific research.

(1) Tourism and leisure

LLNWP is rich in resources and beautiful scenery, and the special natural conditions have created its tourism function. According to the travel cost method [22], the tourism and leisure value is composed of annual direct tourism income, travel expenses, and travel time value. The equation is as follows:

(14) $$V_{t}=F_{1}+F_{2}+F_{3}$$

where *V_t_* represents the annual total value of tourism (CNY/a); *F*_1_ represents annual direct tourism income (CNY/a); *F*_2_ represents the annual travel expenses (CNY/a); and *F*_3_ represents the annual travel time value (CNY/a).

According to the questionnaire survey of the Lotus Lake National Wetland Park Management Center, the number of tourists received by the LLNWP in 2021 was 3 × 10^5^, and the direct income was 8.1 × 10^6^ CNY/a. Of this direct income, ticket income represented 4.456 × 10^6^ CNY, tourism commodity income represented 643,450 CNY, and the annual parking fee represented 3 × 10^6^ CNY. The average parking fee is 10 CNY/d per person, and the average travel time is 1 day. The average daily board and lodging expenses are 10 CNY, and the average daily transportation expenses are 20 CNY. Therefore, the travel expenses, including transportation expenses and board and lodging expenses, are 9 × 10^6^ CNY/a. The value of annual travel time is the product of the opportunity cost of salary per unit of time and the total travel time. This is estimated at 120 CNY/d per person, which is 3.6 × 10^7^ CNY/a in total.

(2) Education

Education, knowledge, and competencies are intangible values, but humans can directly extract them from ecosystems. The benefit transfer method is used to estimate the average value of the possible value of various educational programs from elementary school to higher education provided by the LLNWP ecosystem per unit area [3]. The equation is as follows:

(15) $$V_{k}=S\times U$$

where *V_k_* is the annual value of wetland education and scientific research value service (CNY/a); *S* is the total area of the LLNWP (hm^2^); and *U* is the scientific research and education value generated by a unit of wetland area (CNY/hm^2^·a).

Wetlands are the natural “basis” of the ecosystem, the gene pool of species, and a natural laboratory for scientific research, providing a platform for research and education. According to the annual statistics of the Tieling Natural Resources Bureau, the value of scientific research and education provided per unit area of wetland in Tieling City is 500.03 CNY/hm^2^·a, and the total area of LLNWP is 776.74 hm^2^.

### 2.5. Land-Use Classification Analysis

**Figure 3:**
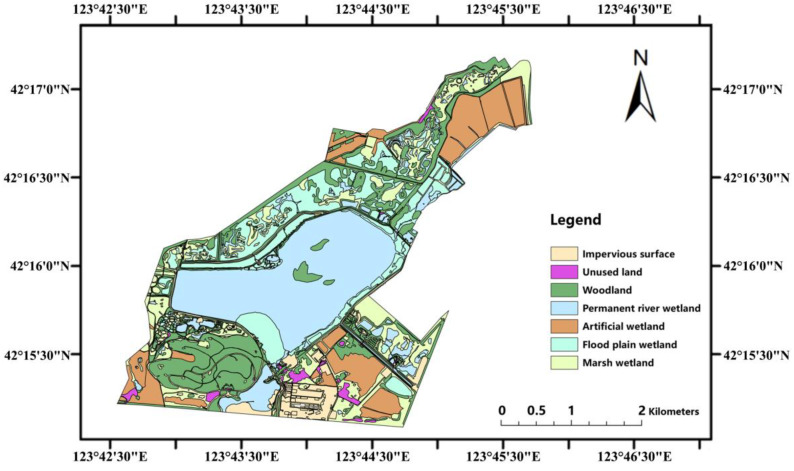
Land-use classification of LLNWP.

According to the field observation data from the ecological station of the LLNWP, and combined with the National Wetland Resource Survey Regulations [13], the land of the LLNWP can be divided into three categories: natural wetlands, artificial wetlands, and other lands. Each category can be further divided into 2–3 levels. Based on the characteristics of the land, the Landsat OLI image of the study area in August 2021 (spatial resolution 15 m) was interpreted using ArcGIS [35], and the LLNWP was divided into seven types of land-use, including permanent river, flood plain wetland, marsh swamp, artificial wetland, impervious surface, unused land, and woodland. The area classified as wetland was taken as the wetland study area (Figure 3). Through calculation, the total size of the study area was 776.74 hm^2^, of which the natural wetland area was 351.62 hm^2^, accounting for 45.27% of the total, and the artificial wetland area was 182.75 hm^2^, accounting for 23.53% of the total (Table 3).

The permanent river wetland in the study area covered the largest size, with an area of 208.97 hm^2^, accounting for 26.9% of the total; the woodland area of 169.1 hm^2^ was the second largest, accounting for 21.77%; the third-largest was the flood plain wetland area of 116.37 hm^2^, accounting for 14.98%. The area of other land-use types in descending order was paddies, with an area of 94.54 hm^2^, accounting for 12.17%; the area of reservoirs and ponds was 88.21 hm^2^, accounting for 11.36%; the herbaceous swamp area was 23.7 hm^2^, accounting for 3.05%; and the forest swamp area was 2.59 hm^2^, accounting for 0.33%.

## 3. Results

### 3.1. Wetland Ecosystem Services Valuation 

#### 3.1.1. Ecosystem Services Valuation of Different Land Types 

**Figure 4:**
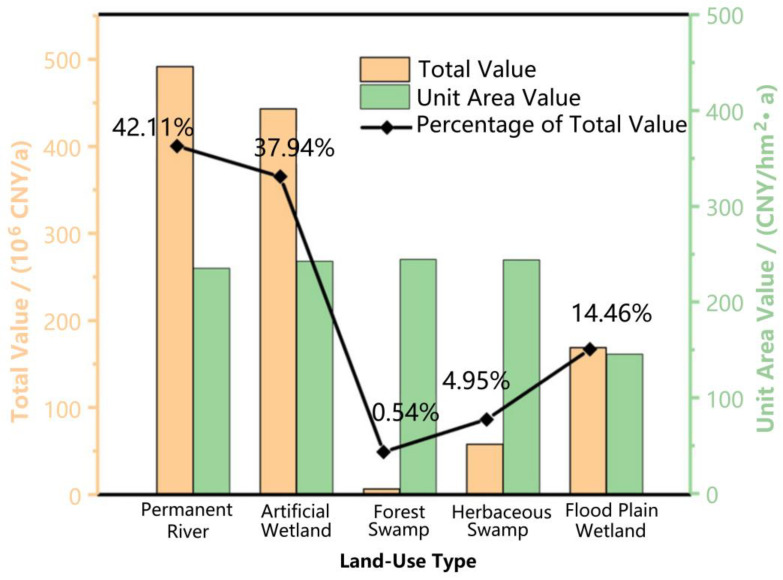
The total and unit area values of functions of ecosystem services in different land-use types.

**Figure 5:**
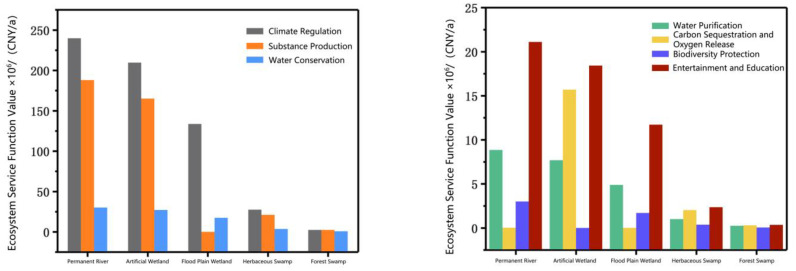
Comparison of the value of each service function in different land-use types.

After calculation, the valuation results of the ecosystem services function in the LLNWP are shown in Table 4. In 2021, the total value of ecosystem services in the LLNWP was 1167.60 × 10^6^ CNY/a, and the unit area value was 218.5 × 10^4^ CNY/hm^2^·a. The value of permanent river reached 491.62 × 10^6^ CNY/a, accounting for 42.11% of the total; the artificial wetland reached 443 × 10^6^ CNY/a, accounting for 37.94%; the forest swamp was 6.31 CNY/a, accounting for 0.54%, the herbaceous swamp was 57.79 CNY/a, accounting for 4.95%; and the flood plain wetland was 168.86 CNY/a, accounting for 14.46%. Furthermore, the unit area value of forest swamp was 244.57 × 10^4^ CNY/hm^2^·a, the herbaceous swamp was 243.84 × 10^4^ CNY/hm^2^·a, the artificial wetland was 242.41 × 10^4^ CNY/hm^2^·a, and the permanent river was 235.26 × 10^4^ CNY/hm^2^·a (Figure 4). In summary, the value of each service function in different land-use types is shown in Figure 5.

#### 3.1.2. Crucial Ecosystem Services Valuation

**Figure 6:**
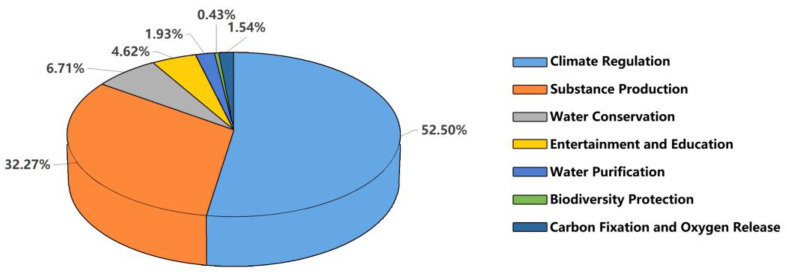
Percentage of each service function value.

**Figure 7:**
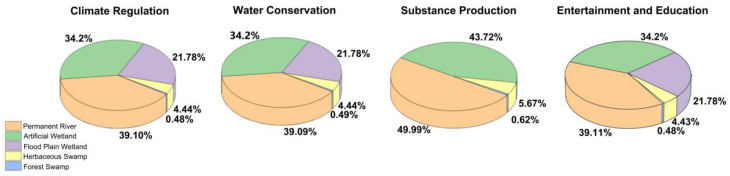
Percentage of different land-use types’ contribution to each service function.

By calculating the ecosystem service valuation of the LLNWP in 2021, climate regulation was found to be the core service function of the study area, with the highest value. This was followed by substance production and water conservation. The remaining function values, in descending order, were entertainment and education, biodiversity protection, carbon fixation and oxygen release, and water purification. The sum of climate regulation, substance production, and water conservation accounted for 91.48% of the total value. Climate regulation accounted for more than half, at 52.50%; substance production accounted for 32.27%; water conservation accounted for 6.71%; and entertainment and education accounted for 4.62% (Figure 6). The four ecosystem service functions occupied a dominant position in the natural functions of the LLNWP, as shown in Figure 7. Permanent rivers had the highest ecosystem services function value with the largest contribution rate to climate regulation, accounting for 39.10%. The other wetlands contributed in descending order from artificial wetland, flood plain wetland, herbaceous swamp to forest swamp, accounting for 34.20%, 21.78%, 4.44%, and 0.48%, respectively. 

#### 3.1.3. The Ecological Service Functions and Socio-Cultural Service Functions Provided by Each Wetland

**Figure 8:**
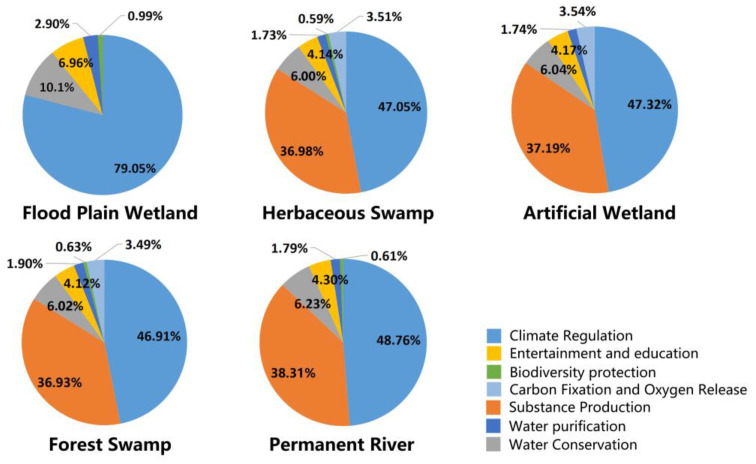
Proportion of various ecological service functions in various types of wetlands.

According to the data analysis, the characteristics of the functions of the ecosystem services of the LLNWP can be classified as follows. Ecological functions include substance production, water purification, water conservation, carbon fixation and oxygen release, climate regulation, and biodiversity protection; socio-cultural functions are entertainment and education [36]. Figure 8 shows the proportion of ecosystem services in each type of wetland, and it is clear the ecological functions of each type of wetland work well, accounting for over 90%; however, socio-cultural functions were seriously lacking in the LLNWP. Therefore, the LLNWP should improve its entertainment and education functions through reasonable planning and design to maximize its advantages.

Permanent river wetlands, flood plain wetlands, herbaceous swamps, forest swamps, and constructed wetlands provided entertainment and education function values of 21.12 × 10^6^ CNY/a, 11.76 × 10^6^ CNY/a, 2.39 × 10^6^ CNY/a, 0.26 × 10^6^ CNY/a, and 18.47 × 10^6^ CNY/a, respectively. Furthermore, the entertainment and education function accounted for 4.30%, 6.96%, 4.14%, 4.12%, and 4.17% of the five types of wetlands. In summary, permanent river wetlands, floodplain wetlands, and artificial wetlands provided more socio-cultural functions, while herbaceous swamps and forest swamps were weaker in this regard. 

### 3.2. Planning and Management of LLNWP

#### 3.2.1. Planning Suggestions

The results of the ecosystem services valuation in the LLNWP clarify the total value, unit area value, and service types of each type of land, combined with the classification of ecosystem service functions. In addition, this demonstrates that the land space of the LLNWP can be reused. We found that forest swamps and herbaceous swamps had the highest ecosystem services function value per unit area. This should strengthen the park’s forest swamp and herbaceous swamp repair and reconstruction planning. As the LLNWP lacks cultural services, this should be enhanced in terms of entertainment and education. The following are planning suggestions based on this:

(1) Construction of shoal wetlands around the permanent river center island

**Figure 9:**
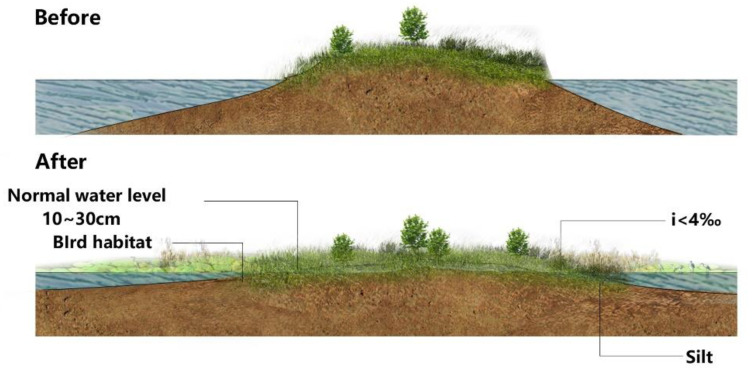
The development of a shoal wetland depicted on a sketch map.

As the park’s permanent river is a part of the reservoir, it must be dredged on a regular basis. The cleaned silt can be utilized to level adjacent open regions with undulating water surfaces, creating an open environment conducive to the establishment of wetland trees, herbaceous growth, and waterbird habitats. In Figure 9, the shoal slope is 4, the width is 5, and the usual water level flooding depth is 10–30 cm [37].

(2) Vegetation restoration in the swamp wetland’s fringe or extension area

**Figure 10:**
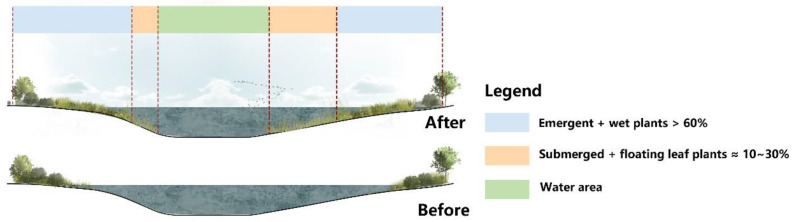
Diagram illustrating vegetation restoration in a marsh wetland.

The wetland vegetation must be regenerated by topographical reform for the conservation and rebuilding of the existing marsh wetland. In addition, native wetland plants must be resistant to low temperatures and freezing damage in the winter, pests and disease, and strong stress. Submerged plants, floating-leaved plants, emergent plants, and wet plants are diverse types of plants dependent on the water gradient. Mixed planting [38] can also be utilized (Figure 10): submerged plant and floating-leaved plant coverage is 10% to 30%, and planting spreads in a random manner alongside an emergent plant and wet plant coverage of 60% planted in a random manner in clusters but not by well-distributed line spacing.

(3) Plant Distribution in Wetlands

**Figure 11:**
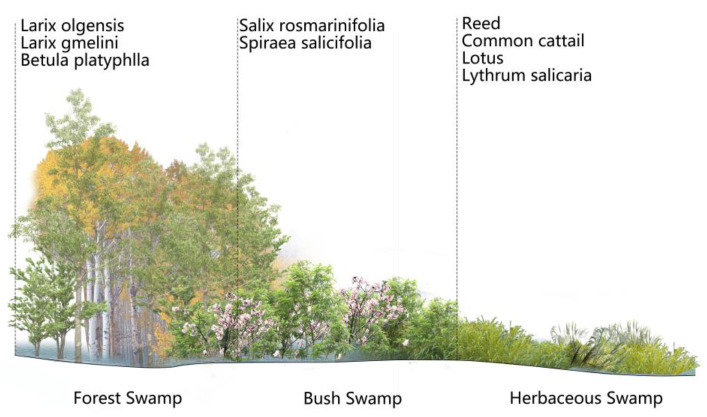
Diagram of a wetland plant layout.

The recommended tree species for restoring the forest wetland are Larix olgensis (*Larix olgensis* Henry and *Larix gmelina* (Rupr) Kuzen) and white birch (*Betula platyphlla* Suk.). Planting ramet subterraneous stems restores the herbaceous marsh, and plants such as reeds (*Phragmites australis* (Cav.) Trin. ex Steud.), cattails (*Typha orientalis* Presl), lotus (*Nelumbo nucifera* Gaertn.), purple loosestrife (*Lythrum salicaria* L.), calamus (*Acorus gramineus* L.), and others are advised. A vibrant wetland plant environment should be created by selecting plants and paying attention to the forms and colors of their leaves, stems, fruits, and trunks. A wetland plant ecological landscape should also be created with plant heights that are in tune with the surrounding environment and hidden shelters and food sources for wild animals. Simultaneously, a shrub swamp can be built in the transition zone between forest and herbaceous swamp [39,40], for example, using shrubs such as marsh willow (*Salix rosmarinifolia* L. Var brachypoda (Traktv. Et Mey) Y. L. Chou) and willow spiraea (*Spiraea salicifolia* L.), as illustrated in Figure 11.

(4) Enhancement of socio-cultural functions in the LLNWP

The LLNWP includes four types of ecosystem services: regulating services, cultural services, supporting services, and provisioning services. Regulating, supplying, and supporting services are all ecological functions, whereas cultural services activities are socio-cultural. According to calculations and analyses, the ecological services function is greater than the socio-cultural services function. Therefore, it should be enhanced by identifying locations with potential cultural roles. The attraction of the wetland park should be improved by planning after studying land-use and the ecosystem services’ function value.

**Figure 12:**
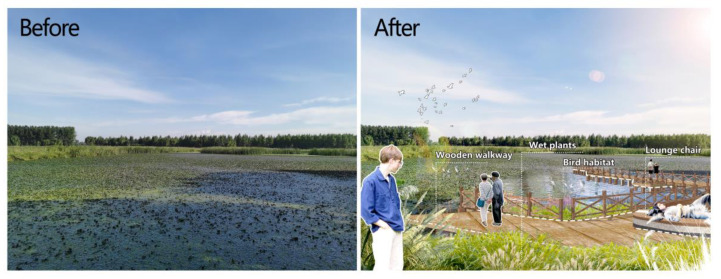
Effects before and after the reconstruction of an artificial wetland.

**Figure 13:**
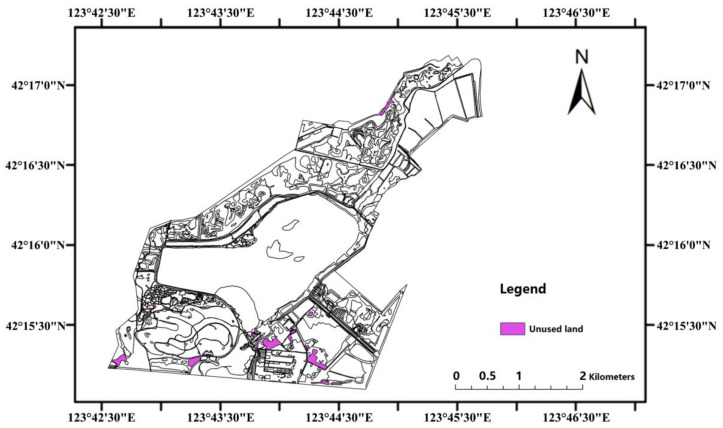
Unused land in LLNWP.

Through the LLNWP’s ecosystem services valuation, it was found that permanent river wetlands, floodplain wetlands, and artificial wetlands have the potential to provide socio-cultural functions. This rebuilds the functional regions without destroying the original ecological purposes, enlarging the leisure and teaching space. Simultaneously, the landscape elements are split into water bodies, plants (trees, shrubs, grasses, wetlands, aquatic), animals, and tiny construction, and these aspects are grouped together by adding wetland plant species and facilities to popular scientific areas. Negotiations with local farmers should take place in order to build an ecological compensation mechanism to maximize the usage of rice fields, such as implementing the square-foot garden concept [41], so that city children can experience nature and form a business model. In the entertainment area, the addition of plank roadways and observation platforms could improve the contact between spectators and the water (Figure 12). Wetland creatures should also be taken into account. Some artificial habitats can be built, allowing animals to interact with people in artificial environments. According to the results of the existing land-use analysis, there is a lot of unused land in the LLNWP, so reusing these spaces through artificial wetland construction can make the LLNWP’s wetland ecological services more sound (Figure 13).

#### 3.2.2. Management Suggestions

According to the valuation results of the LLNWP, the ecosystem services function has a higher economic value and plays an important role in the ecological services of this region. This should be fully considered in the protection, utilization, and planning management of the LLNWP. The protection of existing wetland resources should be strengthened, alongside strictly controlling the scale of wetland development, improving relevant laws and regulations on wetland protection, increasing wildlife protection and management, establishing an LLNWP information and monitoring system, strengthening ecological monitoring, and comprehensively treating water pollution. The local government should increase the special financial funds budget for the protection of the LLNWP and increase citizens’ awareness of participation in the protection of the urban wetland. The original wetland should be protected, avoiding excessive exploitation and utilization of the LLNWP. The existing wetland resources should also be integrated to form a complete ecosystem system to promote the common sustainable development of ecology, the economy, and society.

## 4. Discussion

### 4.1. Wetland Ecosystem Services and Human Wellbeing

Wetlands between terrestrial and aquatic ecosystems have special ecological structures and functions. In addition to their effective filtration capacity and richness in biodiversity, wetlands can be directly used by humans to provide some unique ecosystem service functions, such as socio-cultural services. Human wellbeing is significantly related to the surrounding environment, and the quality of wetlands has a great influence on both the mental and physical health of nearby residents. Wetlands’ socio-cultural service functions can increase the quality of life of humans, strengthen their happiness, generate positive emotional responses, recover physical and mental health, and encourage them to engage in appropriate activities, among other things [42].

The wetland ecosystem has the highest value per unit area in the world [3]. Globally, the annual value of various ecosystems per unit area has been researched. The forest ecosystem is 969 CNY/hm^2^·a, the grassland ecosystem is 232 CNY/hm^2^·a, and the farming ecosystem is 92 CNY/hm^2^·a. According to the current exchange rate, the value per unit area of the LLNWP is 150.32 × 10^4^ CNY/hm^2^·a. The wetland ecosystem in the LLNWP has a higher unit area value than the other three ecosystems, which is in line with Costanza’s research on the value of the functions of the ecosystem services.

### 4.2. Wetland Ecosystem Services Valuation

Supporting, provisioning, cultural, and regulating services were identified in the LLNWP ecosystem service functions. Evaluation indicators and methods for value quantification were selected by a thorough examination of the accuracy, convenience, and cost of data acquisition for various function types [43]. Finally, the valuation index system for the LLNWP ecosystem service functions was constructed. The LLNWP evaluation method in this study was appropriate. This method of evaluating and classifying ecosystem services is not just applicable to the LLNWP’s wetland ecosystem but also to forest [44] and grassland ecosystems. The non-interrelation [45] classification of evaluation and the single econometric approach employed in earlier ecosystem studies resulted in duplicate calculation findings and incompatibility with wetland ecosystems, and the methodologies utilized varied widely due to diverse evaluation aims [46,47]. As a result, research outcomes are less comparable [48].

After the evaluation, firstly, the dominant ecosystem service function was climate regulation in the LLNWP. This confirms that wetlands are conducive to climatic stability, thereby providing protection and support for human survival. Secondly, in terms of unit area, the value of the functions of the ecosystem services of different land-use types in descending order are forest swamps, herb swamps, artificial wetlands, permanent rivers, and floodplain wetlands. In line with the law, the greater the biomass, the richer biological species the wetland contains; the smaller the disturbance caused by human planning, the more complete the ecological patches will be, and the more ecological islands will be built, resulting in a higher service value of the wetland. Due to the different main functions under diverse land uses, the LLNWP is divided into natural wetlands and artificial wetlands. Natural wetlands are mainly river and marsh wetlands; artificial wetlands are mainly paddy fields. Comparing the per-unit-area service function value data of a natural wetland and artificial wetland, the natural wetland has formed a relatively stable ecological structure during the long-term success of the ecosystem, which is specifically manifested in complete ecological patches, high biomass, complex biodiversity, and perfect ecological function. In contrast, with greater human disturbance and influence, artificial wetlands (for example, arable land, reservoirs and ponds) have the disadvantages of a simple structure, a single functionality, and low biodiversity.

When estimating each indicator, the geographical and resource characteristics of the study area were combined. There were some flaws in the research methodology. For example, the humidification of wetlands mainly includes water evaporation and plant transpiration in open water and vegetation-covered areas. The soil covered by wetland vegetation has lower sunlight reflectivity, and the effect of warming is more obvious than on water bodies. Consequently, gaps similar to capillaries are formed between soil particles, and the uneven surface also greatly increases the evaporation area and amount. At the same time, the wetland plants growing above also increase the surrounding humidity through their transpiration. However, the plant transpiration ability is weak, limited by the high latitude of the LLNWP, brief periods of high temperatures, and insufficient solar radiation. Therefore, this study ignored the humidification effect of wetland plant transpiration on the surrounding environment when calculating the humidification value and only calculated the cost of electricity for water evaporation. “Biodiversity” is the sum of the ecological complex formed by animals, plants, microorganisms, the environment, and the various ecological processes related to it. Since the ecological value of plant biodiversity is mainly manifested in carbon fixation and oxygen release, water purification, climate regulation, etc., the value of microorganisms is difficult to evaluate. Furthermore, birds account for as much as 75.85% of animal resources. Therefore, the biodiversity valuation in this paper primarily calculated bird conservation and habitat values. The conservation of zoobenthos, on the other hand, serves an essential role in the wetland environment. As a result, the animals and plants in wetlands should be further examined and analyzed, and the value accounting for biodiversity will become more precise in the future.

### 4.3. Wetland Park Planning and Management

Human wellbeing can be enhanced through appropriate environmental modifications. The most direct approach to connect humans and natural ecosystems is to use wetlands. The wetland type has a direct relationship with the ecosystem’s structural integrity, value function type, and value level. It may provide certain data to support wetland park landscape planning and management based on the diverse ecological values created by different wetlands. Landscape planning is an essential method of enhancing the living environment. Wellbeing can be constructed by reasonably utilizing land space and environmental resources. Wetland parks are a common form of wetlands in cities. The contribution of the wetland ecosystem to human wellbeing can be increased under the protection of pertinent legislation, via its design, and through its management. Landscape planning is the modification of the ecosystem to fulfill human requirements, whereas ecosystem services are the benefits that humans gain from the ecosystem. Both humans and ecosystems are used as research subjects. Scholars have typically conducted specialized research on a single service, such as rain and flood management [49], biological control [50], landscape aesthetics, and recreation [51]. Ecological landscape planning has as its goal and foundation the promotion of the value of the functions of ecosystem services, making it an effective strategy for ensuring the production and effectiveness of ecosystem services [52]. With the current state of the wetland landscape, a planning and design approach based on ecosystem services valuation was proposed. Landscape planning for the LLNWP was combined with land cover to divide the space functions, which was based on clarifying wetland types, defining their spatial distribution, and evaluating ecosystem services.

Ecosystem service functions are crucial characteristics of wetlands, and they are affected by geographic environments. The local climate and species habitat environment in the LLNWP were taken into account in landscape planning that conformed to the relevant research results [53]. Moreover, the enhancement of the cultural function of a wetland park can drive the local tourism economy [54]. The research region is connected to metropolitan residential areas, and the most important cultural services are entertainment and education. Therefore, planning and designing based on land type analysis employ incrementally smaller facilities [55] and plant species. The square-foot garden concept was adopted to encourage man–nature interaction and to maximize the available cultural space. For better planning and management, the relationship between ecosystem services and landscape planning has been established through land space.

## 5. Conclusions

Human wellbeing is significantly related to the quality of the surrounding wetlands. The LLNWP plays an important role in the regional economic development and environmental protection of Tieling City. The value of wetlands to human wellbeing is determined by their contribution to the quality of life of the surrounding residents, so their huge ecological benefits can provide a more suitable and healthier environment and improve the quality of life for human beings. In 2021, the total value of ecosystem services in the LLNWP was 1167.60 × 10^6^ CNY/a, and the average unit area value was 218.5 × 10^4^ CNY/hm^2^·a. The wetland ecosystem in the LLNWP had a higher unit area value than the other three ecosystems. The permanent river and artificial wetlands had a high ecological service value; the forest swamps and herbaceous swamps had a high unit area value, and climate regulation was the core ecological service function. However, the socio-cultural function was relatively weak.

This study has extended the existing research and achieved the high provision and regulation of ecosystem services through spatial land use, which establishes a link between ecological function and landscape planning. The land space can be reused through landscape planning to maximize the service function benefits of the wetland ecosystem, including improving ecological service functions through the restoration of swamp wetland vegetation or land leveling and improving socio-cultural service functions through the rational use of idle land in artificial wetlands or the addition of diminutive facilities and water platforms. This can increase the total value of urban wetlands by identifying and revealing their value.

Wetlands also have a unique and non-negligible role in ecosystem services and human wellbeing. Reasonable landscape planning combines valuation with land analysis, which is conducive to the sustainable development of wetland tourism. To construct better wetland landscape spaces for human welfare, more accurate remote sensing technology and biological monitoring data should be combined to improve the accuracy of value calculations in future research.

## Figures and Tables

**Table 1:** Data source of ecosystem services valuation in the Lotus Lake National Wetland Park (LLNWP).

Type	Data Category	Value	Data Source
Substance resource	Rice average market price	2830 CNY/t	*Tieling City’s Statistical Yearbook, 2020* (http://www.tieling.gov.cn/, accessed on 26 December 2021) [16]
	Reed average market price	600 CNY/t	
	Lotus average market price	3000 CNY/t	
	Water average market price	3.2 CNY/t	
	Annual unit rice yield	8.6249 t/hm^2^·a	
	Annual unit reed yield	10.3124 t/hm^2^·a	
	Annual unit lotus yield	48.749 t/hm^2^·a	
	Annual unit water yield	540,007.8 t/hm^2^·a	
	Rice area	94.54 hm^2^	Field surveys and the remote sensing image interpretation in September 2021, LLNWP
	Reed area	23.7 hm^2^	
	Lotus area	88.21 hm^2^	
	Water area	208.97 hm^2^	
Water purification	Frost-free period	165 d	Tieling Meteorological Bureau, 2021
	Wastewater treatment amount	5 × 10^4^ t/d	China Economic and Social Development Statistics Database (https://data.cnki.net/, accessed on 15 June 2021) [17]
	Wastewater treatment cost	2.73 CNY/t	
	Porosity	33%	Tieling Natural Resources Affairs services Center, 2021
	Hydraulic conductivity	38%	
	Chemical oxygen demand removal rate	18.2%	Field surveys and measurements in September 2021, LLNWP
	Total phosphorus removal rate	85.7%	
	Ammonia nitrogen content removal rate	87.68%	
	Dissolved oxygen average concentration	2.84 mg/L	
Water conservation	Average water level line	54 m	Water Resources Bulletin of Tieling City, Liaoning Province, 2021 (https://slt.ln.gov.cn/jbgb/szygb/, accessed on 25 September 2021) [18]
	Water storage cost	0.67 CNY/m^3^·a	
Carbon fixation and oxygen release	Precipitation	6920 mm/a	Tieling Meteorological Bureau, 2021
	Total solar radiation	5.049 × 10^5^ J/cm^2^·a	
	Average temperature	8.75 °C	
	Swamp plant area	230.88 hm^2^	Field surveys and the remote sensing image interpretation in September 2021, LLNWP
	Rice economic coefficient	0.35–0.6	Shenyang Agro-ecosystem Experimental Station, Chinese Academy of Sciences, 2021
	Moisture content of typical rice grains	13.5	
	Rice underground biomass	55 g/m^2^·a	
	Rice carbon coefficient	0.47	
	China’s afforestation costs	250 CNY/t	National Forestry and Grassland Administration (https://www.forestry.gov.cn/, accessed on 21 August 2021) [13]
	Unit industrial oxygen production cost	0.4 CNY/kg	China Economic and Social Development Statistics Database (https://data.cnki.net/, accessed on 15 June 2021) [17]
Climate regulation	Air-conditioning average energy efficiency ratio	3.4	Field surveys and *Tieling City’s Statistical Yearbook*, *2020* (http://www.tieling.gov.cn/, accessed on 26 December 2021) [16]
	Each air-conditioning heat absorption	432,804.2 J/d	
	Local electricity bill	0.5 CNY/kW·h	State Grid Tieling Power Supply Company, 2021
	Wetland area	534.39 hm^2^	Field surveys and the remote sensing image interpretation in September 2021, LLNWP
	Humidifier electricity consumption to convert unit water	125 kW·h/m^3^	Field surveys and *Tieling City’s Statistical Yearbook*, *2020* (http://www.tieling.gov.cn/, accessed on 26 December 2021) [16]
	Average evaporation	1262 mm	Water Resources Bulletin of Tieling City, Liaoning Province, 2021 (https://slt.ln.gov.cn/jbgb/szygb/, accessed on 25 September 2021) [18]
	LLNWP total area	776.74 hm^2^	Field surveys and the remote sensing image interpretation in September 2021, LLNWP
Soil retention	Soil average nitrogen content	1.16 g/kg	Tieling Environmental Protection Monitoring Station, 2021
	Soil average phosphorus content	0.05 g/kg	
	Soil organic matter content	2.01 g/kg	
	Phosphorus content of diammonium phosphate fertilizer	460 g/kg	*Tieling City’s Statistical Yearbook, 2020* (http://www.tieling.gov.cn/, accessed on 26 December 2021) [16]
	Nitrogen content of diammonium phosphate fertilizer	180 g/kg	
	Diammonium phosphate fertilizer price	2.1 CNY/kg	
	Organic matter price	0.32 CNY/kg	
Biodiversity protection	The number of first-level protected birds	17	Tieling Natural Resources Affairs Services Center, 2021
	The number of second-level protected birds	69	
	The price of first-level protected birds	135,072.50 CNY/pcs	The “willingness to pay for protected birds” of residents in the Tieling area in 2021 [19]
	The price of second-level protected birds	17,309.33 CNY/pcs	
	Wetland ecosystem biodiversity conservation annual unit value	2212.2 CNY/hm^2^·a	Statistics Information Network of Liaoning Province (https://tjj.ln.gov.cn/, accessed on 25 July, 2021) [20]
	Habitat area	703.47 hm^2^	Field surveys and the remote sensing image interpretation in September 2021, LLNWP
Tourism and leisure value	Number of tourists	3 × 10^5^ person	Lotus Lake National Wetland Park Management Center, 2021
	Ticket income	4.456 × 10^6^ CNY/a	
	Tourism commodity income	643,450 CNY/a	
	Average parking fee per person	10 CNY/d	
	Average travel time per person	1 d	
	Average board and lodging expenses	10 CNY/d	
	Average transportation expenses	20 CNY/d	
	Opportunity cost salary per unit time	120 CNY/d	
Culture, education and scientific research	Wetland ecosystem scientific research and education annual unit value	500.03 CNY/hm^2^·a	*Tieling City’s Statistical Yearbook, 2020* (http://www.tieling.gov.cn/, accessed on 26 December 2021) [16]

Note: Except for the websites and related references marked, the data sources were obtained through field investigations and visits to relevant local resources or management departments.

**Table 2:** Evaluation index system for the service performance of LLNWP.

Ecosystem Services	Index Category	Evaluation Index	Evaluation Method
Supporting services	Substance production	Food	Market value
		Raw materials	Market value
		Water resources	Market value
Regulating services	Water purification	Degrading pollutants	Shadow engineering
	Water conservation	Water volume regulation and storage	Shadow engineering
	Carbon sequestration and oxygen release	Plant carbon sequestration	Carbon tax
		Soil carbon sequestration	Carbon tax
		Oxygen release	Shadow engineering
	Climate regulation	Temperature regulation	Benefit transfer
		Humidification	Shadow engineering
	Soil retention	Soil retention capacity, nutrient preserving capability	Shadow engineering
Provisioning services	Biodiversity protection	Bird conservation	Market value
		Habitat conservation	Benefit transfer
Cultural services	Entertainment and education	Tourist and leisure	Travel cost
		Culture, education and research	Benefit transfer

**Table 3:** Land-use classification and area in LLNWP.

Level 1	Level 2	Level 3	Area	Total Area of Each Wetland	Proportion
			hm^2^	hm^2^	%
Natural wetland	River wetland	Permanent river wetland	208.97	351.62	45.27
		Flood plain wetland	116.37		
	Marsh wetland	Herbaceous swamp	23.7		
		Forest swamp	2.58		
Artificial wetland	Reservoirs and ponds		88.21	182.75	23.53
	Paddy		94.54		
Others	Woodland		169.1	242.37	31.20
	Impervious surface		49.62		
	Unused land		23.65		
	Total		776.74	776.74	100

**Table 4:** The values of the functions of ecosystem services of LLNWP.

Services Function	Riverine Wetland		Marsh Wetland		Artificial Wetland	Value
	Permanent River	Flood Plain Wetland	Herbaceous Swamp	Forest Swamp		×10^6^ CNY/a
Substance production	188.37		21.37	2.33	164.74	376.81
Water purification	8.81	4.90	1.00	0.12	7.70	22.53
Water conservation	30.61	17.05	3.47	0.38	26.78	78.29
Carbon sequestration and oxygen release			2.03	0.22	15.68	17.93
Climate regulation	239.70	133.48	27.19	2.96	209.63	612.96
Biodiversity protection	3.01	1.67	0.34	0.04		5.06
Entertainment and education	21.12	11.76	2.39	0.26	18.47	54.00
Value ×10^6^ CNY/a	491.62	168.86	57.79	6.31	443.00	1167.60

Note: The erosion effect of soil and water loss during the flat-water period is not strong in this area, and the value of soil retention has not been calculated. However, the wetland can exert a soil retention service function under extremely heavy precipitation conditions.

## Data Availability

The data presented in this study are available on request from the corresponding author.
